# Editorial: Eating Disorders and Weight Disorders: Assessment, Early Diagnosis, Prognosis, Treatment Outcome and the Role of Potential Psychological and Social Factors

**DOI:** 10.3389/fpsyg.2022.924815

**Published:** 2022-06-01

**Authors:** María Angeles Peláez-Fernández, Ana R. Sepúlveda, Emilio J. Compte

**Affiliations:** ^1^Department of Social Psychology, Social Work and Social Services, and Social Anthropology, Faculty of Psychology and Logopedics, University of Málaga, Málaga, Spain; ^2^Department of Biological and Health Psychology, School of Psychology, Autonomous University of Madrid, Madrid, Spain; ^3^Eating Behavior Research Center, School of Psychology, Universidad Adolfo Ibáñez, Santiago, Chile; ^4^Research Department, Comenzar de Nuevo Treatment Center, Monterrey, Mexico

**Keywords:** eating disorders, weight disorders, assessment, early diagnosis, prognosis, treatment, psychological and social factors

We are delighted to introduce a very special Research Topic of Frontiers in Psychology. Eating disorders (ED) and weight disorders (WD) are severe illnesses in which especially, but not exclusively, young people go through critical disturbances in their eating behaviors and related thoughts and emotions. Given the severity and scope of obesity and unhealthy diets on health, these disorders could have serious outcomes on health, quality of life, and global economy if prevention mechanisms are not arranged. Despite the research conducted up to date, ED and WD are a subject of permanent relevance due their life-threatening condition. In this regard, the field is matter of continuous update aiming to increase the knowledge, improve treatment options, incorporate new research methods and technology, reaching understudied populations, and understanding the impact of external factors. This Research Topics therefore aims to be a contribution to the field of ED and WD; moreover, given the situation of the recent pandemic of COVID-19, which its outbreak on depression, anxiety, distress, insomnia, and post-traumatic stress have been explored (de Medeiros Carvalho et al., 2020; Torales et al., 2020), but little is yet known concerning the effects on ED and WD.

This Research Topic includes 16 articles, 13 of them are based on original research coming from different countries (Canada, Chile, China, France, Spain, and Germany). Most of the original research are quantitative studies, and only one study is qualitative (Ziser et al.). There is also a review article (Hampshire et al.) and a study protocol (Quiles et al.). The Research Topic also includes a correction (Marco et al.) of the corresponding study.

Four papers have explored the potential role of psychological resources (emotional intelligence and meaning in life) and psychosocial stressors (parenting styles, social weight stigma) on the development and maintenance of these disorders. Peláez-Fernández et al. carried out an empirical study with 516 Spanish undergraduate students and community adults of both sexes, age ranged 18–77. They found that deficits in emotional intelligence (EI) leads to ED symptomatology both directly and indirectly through a sequential mediating path. Specifically, high levels of EI were associated to greater self-esteem, which subsequently reduces anxiety, thus predicting lower levels of ED symptomatology. Marco et al. estimated the mediating role of meaning in life in the relationship between emotional dysregulation and ED pathology in three Spanish samples with diverse risk (292 participants with ED, 122 with obesity and 156 control). They found that meaning in life acted as a mediator between emotional dysregulation and the main symptoms of ED. These results highlight the relevance of considering meaning in life as a variable in the onset and maintenance of ED. Fan et al. proposed a model to explain the relationship between diverse types of weight stigma and quality of life among children in Hong Kong. They found associations between the weight stigmas and both child-rated and parent-rated quality of life. Also, weight stigma and weight-related self-stigma mediated the association between body weight and children's quality of life. Finally, Hampshire et al. describe in their systematic review, that parenting styles characterized by high levels of control and low levels of responsiveness were associated to disordered eating among young adults such as unhealthy weight control behaviors. The association was frequently indirect and mediated by several underlying mechanisms including emotional reactivity, lowered self-competence and offspring psychological distress. These results suggest that parenting styles, parents' difficulties in managing their emotions, their perceived competence, and their psychological distress influence the development of disorders eating of their young-adult children.

Following the paradigm of *open science* (UNESCO, 2019), Quiles et al. have published their intervention protocol prior to conduct their study. Publications of study protocols ensures transparency in research, and provides a documented record of the research plan of action, detailing in advance the rationale, methodology and analyses. They adapted an intervention program (ECHOMANTRA) to Spanish caregivers and adolescent patients with ED. The intervention is based on emotional regulation and eating behaviors, with an emphasis on behavior change strategies. The objective is to generate cognitive and behavioral changes and strengthen relationships with family and friends to help the transit from inpatient care to daily life and to prevent relapses.

The study by Sierra et al. explores the emotional processing of food images comparing three clinical groups and a normal-weight comparison group. ED patients are characterized by more negative appraisal, less emotional dominance, and a higher level of arousal when processing food-relevant information. Negative affect mediated the relationship between eating symptomatology and emotional processing. This supports the transdiagnostic model of ED, and the use of similar treatments for different conditions.

To generate an exploratory bio-psychological-familial model, the case-control study conducted by Sepúlveda et al. identified some specific correlates associated with the development of an eating disorder. The model showed that patients' drive of thinness and self-oriented perfectionism, together with the decrease of triiodothyronine (T3) hormone and higher levels of anxiety increase adolescents' risk of developing an ED. Both the increase in fathers' emotional overinvolvement, and the decrease in fathers' depression and mothers' anxiety were specifically associated to the onset of an ED.

Using structural equation modeling, Obeid et al. explored a complex model of shared pathways integrating various theoretical facets for eating and weight disorders in a large community-based sample of male and female adolescents. Higher levels of stressors (weight-based teasing, negative life events), dysregulated eating and poor body image all directly influenced higher weight status. It confirmed the concept that socio-environmental factors would be associated with body image and dysregulated eating, mediated by the complex interplay of psychological and behavioral factors.

Among bariatric surgery patients, El Archi et al. explored the psychological factors associated with binge eating and food addiction, and the role of emotion dysregulation, alexithymia and personality dimensions in the association between attention-deficit/hyperactivity disorder and addictive-like eating. Emotion dysregulation, conscientiousness, agreeableness, and neuroticism are total mediators of this association, with alexithymia being a partial mediator. Negative emotion leads to food intake, increasing positive emotion seeking especially if associated with emotion dysregulation, leading to the development of an addiction. Despite the controversies of the concept of food addiction, authors suggested that certain foods may lead to addictive-like eating that could contribute to overeating and obesity.

The influence of the familial context was investigated in the qualitative study of Ziser et al., which explored the barriers to behavior change in a group of 16 parents of preschool children with overweight and/or obesity. They were identified through a primarily inductive content analysis conducted through an interview. The underestimation of health consequences of overweight and obesity, and a deficient awareness on the problem were the main barriers. The use of the motivational interview in the pediatric environment are suggested to address these topics.

Relevant to assessment, three articles aimed to validate measures of ED symptomatology as well as related constructs such as appearance perfectionism and food craving across Hispanic samples. Lizana-Calderón et al. analyzed the psychometric properties of the Eating Disorder Inventory-3 (EDI-3; Garner, 2004) in a large sample of adolescents and young-adult Chilean women and men. Following Brookings et al. (2020), the study sought to assess alternative models including a second-order two-factor model through exploratory structural equation modeling. The retained model presented a two-bifactor structure, with 12 specific factors. In addition, overall internal consistency was adequate suggesting that the EDI-3 is a valid measure to assess ED in Chilean population. On the other hand, Rica et al. presented results from a cross-validation exploratory and confirmatory approach for the validation of the Physical Appearance Perfectionism Scale (PAPS; Yang and Stoeber, 2012) in a representative sample of male college students in Spain. Findings from the Spanish validation replicates the 12-item two-factor structure of the PAPS that assess two dimensions of appearance perfectionism, Hope for Perfectionism (related to positive reinforcement of achieving attractiveness) and Worry about Imperfection (related to attempts to avoid imperfection, disapproval, and criticism). With excellent levels of internal consistency, the validation of the PAPS not only represents a contribution for the study of ED and WD among Hispanics but also for a yet understudied population in the ED field such as men. Finally, in the framework of third-wave therapies such as Acceptance and Commitment Therapy, Manchón et al. conducted two studies to validate a measure of food craving acceptance, The Food Craving Acceptance and Action Questionnaire (FAAQ; Juarascio et al., 2011). A confirmatory factor analysis on the original model showed an inadequate fit in a sample of Spanish undergraduate students. Consequently, items reviewed, and the scale was modified. An exploratory factor analysis in a second community-based sample described a two-factor structure for the Spanish version of the FAAQ (FAAQ-S). Both subscales, Acceptance and Willingness, showed excellent levels of internal consistency suggesting that the FAAQ-S represents a contribution to the assessment of third-wave therapies constructs and for the dissemination of third-wave psychological therapies in Spain.

Finally, two papers aimed to assess the impact of the COVID-19 pandemic on ED and WD symptoms. Gobin et al. studied the impact of the COVID-19 lockdown on eating habits, body image, and social media use among adult women with and without symptoms of Orthorexia Nervosa (ON). Based on the diet-related lifestyle change during the pandemic, among women with symptoms of ON, findings showed an aggravation of disordered eating thoughts and behaviors and suggested that social media could be contributing factor to this exacerbation. Consistently, the study of Corno et al. described a tendency to report a higher frequency of disordered eating behaviors during the COVID-19 pandemic. Weight concerns predicted an overall increase in the frequency of restrictive behaviors, and body dissatisfaction was found to be associated with a self-perceived increase of emotional eating frequency among adult women who were never diagnosed with an ED. Altogether, both studies suggest that lifestyle change due to the COVID-19 pandemic influenced ED and WE symptoms.

A variety of populations from Asia, Europe, North America, and South America including clinical and community-based samples are represented across the studies. Diverse research designs were considered to assess mediational mechanisms underlying ED and WD symptoms, as well as emotional processing of ED-related stimuli, and specific correlates associated with the onset of ED among clinical samples. Additionally, factors associated with binge eating and food addition have been explored in bariatric surgery patients, and qualitative data describes barriers to behavior change among parents of overweight/obese children. This Research Topic also includes the validation of three measures across Hispanic samples, and a study protocol of an intervention aimed to improve the transit of inpatient care to daily life. Finally, two studies described the impact of the COVID-19 pandemic on disordered eating behaviors. We expect that this Research Topic represents a contribution to the ongoing scientific knowledge about ED and WD.

## Author Contributions

All authors listed have made a substantial, direct, and intellectual contribution to the work and approved it for publication.

## Conflict of Interest

The authors declare that the research was conducted in the absence of any commercial or financial relationships that could be construed as a potential conflict of interest.

## Publisher's Note

All claims expressed in this article are solely those of the authors and do not necessarily represent those of their affiliated organizations, or those of the publisher, the editors and the reviewers. Any product that may be evaluated in this article, or claim that may be made by its manufacturer, is not guaranteed or endorsed by the publisher.
